# Association of Long Non-Coding RNA Malat1 with Serum Levels of Interleukin-1 Beta and Vitamin D in Patients with Ischemic Stroke

**DOI:** 10.31661/gmj.v12i.2457

**Published:** 2023-02-26

**Authors:** Mahnaz Bayat, Reza Tabrizi, Mohammad Saied Salehi, Najmeh Karimi, Moosa Rahimi, Etrat Hooshmandi, Niloufar Razavi Moosavi, Nima Fadakar, Afshin Borhani-Haghighi

**Affiliations:** ^1^ Clinical Neurology Research Center, Shiraz University of Medical Sciences, Shiraz, Iran; ^2^ Noncommunicable Diseases Research Center, Fasa University of Medical Science, Fasa, Iran; ^3^ USERN Office, Fasa University of Medical Sciences, Fasa, Iran; ^4^ Department of Neurology, Shiraz University of Medical Sciences, Shiraz, Iran; ^5^ Laboratory of Basic Sciences, Mohammad Rasul Allah Research Tower, Shiraz University of Medical Sciences, Shiraz, Iran

**Keywords:** Long Non-coding RNA, Malat1, Interleukin-1 Beta, Vitamin D, Ischemic Stroke

## Abstract

Background:Previous studies have demonstrated the strong association of inflammatory cytokines and vitamin D (VitD) deficiency and ischemic stroke (IS) pathogenesis. Due to the negative correlation between long non-coding RNA (lncRNA) Malat1 and pro-inflammatory factors we decided to investigate the associations between Malat1 expression with serum interleukin-1β (IL-1β), and VitD levels in IS patients. Materials and Methods:In this cross-sectional study, 63 IS patients were included. We used enzyme-linked immunosorbent assays to evaluate the serum levels of VitD and IL-1β. Malat1 expression was evaluated by the real-time polymerase chain reaction test. The associations between Malat1expression with VitD and IL-1β were analysed with linear regression (Stepwise model) and Pearson’s correlation analysis. Results: The Malat1 expression was inversely correlated with stroke severity (r=-0.25, P=0.043). Stepwise regression analysis showed a significant positive relationship between VitD level and Malat1 expression (Beta=0.28, P=0.02), and also showed a non-significant negative relationship between IL-1β and stroke severity. VitD level showed a positive Pearson correlation with Malat1 (r=0.28, P=0.023) and a negative correlation with IL-1β (r=-0.29, P=0.018) while it could not detect a significantly negative correlation with stroke severity. Conclusion: For the first time the associations between Malat1 expression with IL-1β and VitD in IS patients was analyzed. We found a significant positive relationship between VitD and Malat1. This correlation needs to be investigated with a larger sample size to achieve a strong and reliable association between VitD and Malat1.

## Introduction

After heart disease and cancer, stroke is the leading cause of death.Stroke with a history of long-term or permanent post-stroke disability has received extensive clinical research attention. Inflammation plays a major role in various stages of ischemic stroke. A novel therapeutic target for ischemic brain cells is represented by the neuroinflammatory triangle entailing bursts of reactive oxygen species (ROS), inflammatory cytokines release, and disruption of the blood-brain barrier (BBB) [1].

Optimal management of IS requires rapid assessment of stroke severity using ideal biomarkers within the first hours after stroke. The ideal properties of a stroke biomarker are non or minimally invasive, quick, cost-effective, and without interfering with acute therapies [2][3].

long non-coding RNAs (lncRNAs) are a class of RNA transcripts with more than 200 nucleotides that rarely affect protein encoding, while it has shown essential roles in signaling pathways and gene regulation related to diseases. lncRNA, metastasis-associated lung adenocarcinoma transcript 1 (Malat1), has shown a regulatory role in the pathology of ischemic stroke.

Previous studies have revealed that Malat1 lncRNA could be a potential biomarker for the diagnosis as well as prognosis of atherosclerotic cardiovascular disease [4], cancer [5][6][7][8], multiple sclerosis [9], and sepsis [10]. Different experimental and clinical research has shown the anti-inflammatory and anti-apoptotic roles of Malat1 in the brain [11][12][13].

The downregulation of lncRNA Malat1 in IS patients has been reported in previous studies [12][14]. Ren et al. demonstrated that Malat1 expression was inversely associated with the National Institutes of Health Stroke Scale (NIHSS) score and the expression of pro-inflammatory factors (including TNF-α, CRP, IL-6, IL-22, and IL-8) on the first day after stroke [12]. Fathy et al.

in 2021 also reported the downregulation of Malat1 in IS patients with a negative association with stroke severity [14] The soluble glycoproteins that are produced by microglia, astrocytes, endothelial cells, and neurons in response to damaged brain tissue are cytokines. Cytokines clearly play a key role in the pathophysiology of stroke, and a high ratio of pro-inflammatory to anti-inflammatory cytokines is associated with larger infarct volume and poorer functional outcomes in IS patients [15].

Eleven isoforms have been observed in the IL-1 family, out of which, IL-1β plays a strong pro-inflammatory role in ischemic injury and other neurodegenerative diseases [16][17]. IL-1β mediates brain injury through multiple mechanisms following ischemic insult [1]. A complex of IL-1β with transmembrane receptors triggers intracellular signaling pathways including the NF-kB, the c-Jun N-terminal kinases (JNKs), and the p38 mitogen-activated protein kinase (P32 MAPKs). The final effects of these signaling pathways are displayed in the form of more inflammatory damage in the brain [18][19][20].

IL-1β antagonist limited excitotoxicity in damaged brain tissues of rats [21]. Wang et al found a negative association between serum levels of vitamin D and IL-6 in IS patients [22]. According to the previous research, interleukin-1β was significantly increased in the peripheral blood of IS patients compared to the control group [23][24][25]. Recently, an important relationship between the pathogenesis of neurodegenerative disorders and Vitamin D deficiency has been reported [26][27][28][29][30]. Vitamin D has shown a key adjusting role in inflammation and immune response [31], membrane antioxidant activity [32], and also in synaptic plasticity [33].

It has been revealed that serum vitamin D levels in patients with stroke is lower than in healthy subjects which were inversely correlated with stroke severity and functional outcome [34][22].

Vitamin D3 supplementation reduces pro-inflammatory mediators and brain damage in stroke individuals [22][35][36] and ischemic animals [37]. The severity of Vitamin D deficiency could predict the risk of stroke [38], mortality [39], and poor functional outcome [40].

The significant associations between VitD level, Malat1 expression, IL-1β, and NIHSS within the first hours after stroke will provide a better and deeper understanding of the role of Malat1 expression and the protective role of VitD in stroke pathogenesis. Different studies reported the role of cytokines, different lncRNAs, and vitamin deficiency role in brain damage, but the associations between them have rarely been investigated after stroke.

Due to the high prevalence of vitamin D deficiency among the Iranian population, which is one of the major public health issues [41], this study initiated a longitudinal analysis to assess the associations between lncRNA Malat1 with VitD, and serum levels of IL-1β in the peripheral blood of IS patients for the first time.

## Material and methods

Participants

This cross-sectional study was performed in Shiraz Namazi Hospital from August 2020 to 2021. Sixty-three patients who were hospitalized within the first 24 hours after IS were included in the study.

Ischemic stroke in patients 18 years and older, was diagnosed by a neurologist and confirmed by brain non-contrast computed tomography (CT), or diffusion-weighted magnetic resonance imaging. Ischemic stroke is an acute neurologic disorder lasting more than 24 hours [42].

Patients with immunosuppressive therapy, transient ischemic attack, and severe inflammation were excluded from this study. Using the NIHSS score, stroke severity is assessed at admission, with higher scores indicating greater severity [43].

In this study, hypertension and diabetes were diagnosed according to defined criteria [44][45]. The local Ethics Committee of Shiraz University of Medical Sciences has approved the study ethically with grant number (IR.SUMS.REC.1398.17988). Written informed consent was provided by all patients (or their proxy respondents). Peripheral venous blood samples were collected from patients 0-24 hours after the stroke.

Laboratory tests

The coagulated blood was centrifuged (3000 g, 10 minutes), and the serum was stored at −80 °C until use. The major circulating metabolite and the best indicator of vitamin D are 25(OH)D [46]. 25-OH vitamin D levels in the serum of the patients were measured with an enzyme-linked immunosorbent assay (ELISA) Kit (Monobind Inc.®, United States). Serum levels of IL-1β were measured using specific ELISA kit (Kermania Pars Gen, Kerman, Iran) according to the manufacturer’s instructions.

RNA extraction and real-timepolymerase chain reaction

A total RNA extraction kit (Favorgen, Taiwan) was used to extract total RNA from whole blood according to the manufacturer’s instructions. RNA samples with the A260/A230 and A260/A280 ratios above 1.7 were used for cDNA synthesis by cDNA synthesis Kit (AddBio, Korea).

We also used Quantstudio 3 Real-Time PCR System (Applied Biosystems, Foster City, USA) and RealQ Plus 2x Master Mix Green Low Ampliqon, Denmark). The thermal-cycling set was adjusted at 95 -10 min which was accompanied by 40 cycles for 15 s at 95 °C and 1 min at 60 °C and, also in the melting phase thermal setting as follows 15 s at 95 °C, 30 s at 60 °C, and 15 s at 95 °C.

The following primers were used;

Malat1:

-Forward:5′-TCAGTGTTGGGGCAATCTT-3′

-Reverse:5′-CGTTCTTCCGCTCAAATCC-3

TATA box-binding protein (TBP, reference gene):

-Forward:5′-CCCGAAACGCCGAATATAATC-3′

-Reverse:5′-TCTGGACTGTTCTTCACTCTTG-3′

Using the cycle threshold (Ct), the variation of the value in expression levels was analyzed. The difference Ct between TBP and Malat1 was expressed as ΔCt. Finally, 2−ΔCt was used to define the relative Malat1 expression levels for every subject [47].

Statistics

The correlation between different clinical and laboratory parameters was analyzed by the Pearson correlation test. To compare the blood level of Malat1, IL-1β, and VitD in different subgroups of IS patients, we used subgroup analysis with an independent two-sample t-test.

The relationships between lncRNA Malat1, VitD level, IL-1β, and atherosclerotic risk factors were analyzed by a Linear regression (stepwise model) after adjusting the important variables.

The analyses were performed using the SPSS Inc., Chicago, IL, USA (version 19.0) and GraphPad Prism (version 5.01). The P<0.05 was considered statistically significant.

## Results

**Table:** Table1. Demographic and Clinical Characteristics of Ischemic Stroke Patients

Characteristics	IS patients (n=63)	Characteristics	IS patients (n=63)
Male, n (%)	43 (68.3%)	BUN, mg/dL	16.26±5.593
Female, n (%)	20 (31.7%)	Cr, mg/dL	1.20±0.4
Age, years	64.4±1.7	AST (U/L)	21.06±8.807
BMI, (kg/m^2^)	26.39±0.66	ALT (U/L)	18.52±9.959
Hypertension, n (%)	34 (54%)	IL1β, pg/ml	53.37±5.144
Diabetes, n (%)	23 (36.5%)	Malat1(fold change)	3.64±0.643
Hyperlipidemia, n (%)	21 (33.3%)	VitD, ng/ml	23.01±1.482
Smoking, n (%)	10 (15.9%)		
Drinking, n (%)	2 (3.2%)	Types of stroke	
TG, mg/dL	125.4±6.78	LAA	26 (41.2%)
TC, mg/dL	162.04±5.349	SVD	20 (31.7%)
LDL, mg/dL	98.38±4.383	CE	10(15.8%)
HDL, mg/dL	33.8±0.9	UD	7(11.11%)
WBC	7849.21±247.071	NIHSS at admission	
Hgb, (g/dL)	14.03±3.271	≤6	25 (39.7%)
PLT	196301±7274	≥7	38 (60.3%)

Data were shown as mean±SEM or as n (%). **IS:** Ischemic stroke; **BMI:** Body mass index; **TG:** Triglyceride; **TC:** Total cholesterol; **LDL:** Low density lipoprotein; **HDL:** High density lipoprotein; **WBC:** White blood cell; **Hgb:** Hemoglobin; **PLT:** Platelet; **BUN:** Blood urea nitrogen;**Cr:** Creatinine; **AST:** Aspartate aminotransferase ; **ALT:**Alanine aminotransferase; **IL-1β:** Interleukin-1β; **Malat1:** Metastasis-associated lung adenocarcinoma transcript 1; **VitD:**Vitamin D; **LAA:** Large artery atherosclerosis; **SVD:** Small vessel disease; **CE:** Cardiac embolism; **UD:** Undetermined; **NIHSS:** National institutes of health stroke scale.

**Table:** Table2:Pearson Correlation of Vitamin D (VitD), Metastasis-Associated Lung Adenocarcinoma Transcript 1(Malat1) Interleukin-1β (IL-1β) with Clinical Parameters and Together in Ischemic Stroke (IS) Patients.

	Malat1		IL-1βr		VitDr	
	r	P-value	r	P-value	r	P-value
Age	0.206	0.105	-0.019	0.883	0.155	0.226
BMI	-0.084	0.513	0.095	0.459	-0.281	0.026
FBS	-0.058	0.65	-0.033	0.797	-0.028	0.827
PLT	-0.071	0.579	0.123	0.337	-0.208	0.102
WBC	-0.218	0.086	0.277	0.028	-0.166	0.193
HB	0.111	0.385	-0.049	0.706	0.031	0.811
HDL	0.043	0.74	0.105	0.411	-0.043	0.741
LDL	-0.049	0.705	0.063	0.626	-0.158	0.215
TC	0.01	0.938	0.066	0.606	-0.121	0.346
TG	-0.158	0.216	-0.136	0.287	-0.091	0.476
ALT	-0.021	0.868	0.055	0.669	0.062	0.630
AST	-0.075	0.56	-0.018	0.888	-0.129	0.312
Cr	0.189	0.137	-0.215	0.09	0.067	0.6
BUN	0.077	0.549	0.011	0.934	0.31	0.013
NIHSS	-0.256	*0.043	0.584	***0.0001	-0.223	0.08
Malat1			-0.28	*0.027	0.287	*0.023
IL-1β	-0.28	*0.027			-0.296	*0.018
VitD	0.287	*0.023	-0.296	*0.018		

**BMI:** Body mass index; **FBS:** Fast blood sugar; **PLT:** Platelet; **WBC:**Wight blood cell; **HB:** Hemoglobin; **HDL:** High-density lipoprotein; **LDL:** Low-density lipoprotein; **TC:** Total cholesterol; **TG:** Triglyceride; **ALT:** Alanine aminotransferase; **AST:** Aspartate aminotransferase; **Cr:** Creatinine;**BUN:** Blood urea nitrogen; **NIHSS:** National institutes of health stroke scale; **Malat1:** Metastasis-associated lung adenocarcinoma transcript 1; **IL-1β:** Interleukin-1β; **VitD:** Vitamin D. (*P<0.05, ***P<0.001).

**Table:** Table3:Comparison of Metastasis-Associated Lung Adenocarcinoma Transcript 1(Malat1) Interleukin-1β, (IL-1β) Malat1, IL-1β and Vitamin D (VitD), in Different Subgroups of Ischemic Stroke (IS) Patients

	Malat1	P	IL-1β (pg/ml)	P	VitD (ng/ml)	P
Sex						
Male	3.9 ±0.85	0.35	54.9 ±6.2	0.36	23.2 ±1.7	0.81
Female	3.0 ±0.88		53.4 ±9.3		22.3 ±2.6	
Diabetes						
Positive	3.4 ±0.97	0.92	63.9 ±9.1	0.31	21 ±1.9	0.32
Negative	3.7 ±0.85		48.8 ±6.1		24 ±2	
Hypertension						
Positive	4 ±0.7	0.52	52.5 ±7.1	0.75	22.1 ±1.8	0.47
negative	3.1 ±1		56.8 ±7.6		23.8 ±2.3	
Hyperlipidemia						
Positive	4 ±0.99	0.66	51.1 ±8.9	0.34	22.1 ±2.4	0.93
negative	3.4 ±0.83		56.2 ±6.4		22.9 ±1.8	
Smoking						
Positive	3.9 ±1.4	0.96	46.4 ±10.7	0.26	25.3 ±3	0.73
negative	3.5 ±0.7		56.0 ±5.8		22.5 ±1.6	
Drinking						
Positive	4.5 ±3.3	0.86	82.3 ±47.8	0.37	18.5 ±1.5	0.25
negative	3.5 ±0.65		53.6 ±5.2		23 ±1.5	
NIHSS						
(admission)	5±1.3	**0.001	35.1 ±2.9	***0.000	24.2 ±2.5	0.7
≤0-6	2.7 ±0.51		65.3 ±7.7		22.1 ±1.7	
>7						

**NIHSS:**National institutes of health stroke scale; **Malat1:**metastasis-associated lung adenocarcinoma transcript 1; **IL-1β:**Interleukin-1β; **VitD:**Vitamin D; (**P<0.01 and ***P<0.001)

The expression level of Malat1 lncRNA and the serum level of IL-1β, and VitD in IS patients 63 IS patients with a mean age of 64.4±1.7 years (minimum: 28 and maximum: 90 years) were included in this study. The mean of Malat1 in the peripheral blood of IS patients was evaluated by RT.PCR and reported 3.64±0.6 (fold change). The mean level of IL-1β and VitD in the serum of patients were measured as 53.37±5.14 (pg/ml) and 23.01±1.4 (ng/ml) respectively (Table-1).

Pearson correlation of VitD, Malat1, IL-1β with clinical parameters in IS patients

The Pearson correlation test showed that the Malat1 expression had a significant positive correlation with VitD level (r=0.28, P=0.023) and a significant negative correlation with IL-1β level in our patients (r=-0.28, P=0.027) (Table-2). The NIHSS score has shown a significant negative correlation with the Malat1 level (r=-0.25, P=0.04) and also a positive correlation with IL-1β (r=0.58, P=0.0001). A non-significant negative correlation was also detected between VitD level and NIHSS score (r=-0.22, P=0.08).

Comparison of the blood level of Malat1,IL-1β, and VitD in different Subgroups of IS patients

Subgroup analysis showed that sex, hypertension, hyperlipidemia, diabetes, smoking, and drinking did not affect Malat1 expression, IL-1β, and VitD in peripheral blood of ischemic stroke patients (Table-3).

The evaluation of stroke severity in IS patients revealed that the Malat1 expression significantly was lower (2.7±0.51 vs 5±1.3, P=0.001) in patients with high NIHSS score (>7), while IL-1β was higher in patients with NIHSS 0-6 (65.3±7.7 vs 35.1±2.9, P=0.000).

Relationship between Malat1 expression with VitD, IL-1β, and atherosclerotic risk factors in IS patients by Linear regression (stepwise model)

After adjusting the important variables, stepwise regression analysis showed that VitD level could only show a significant positive relation with the expression level of Malat1 (Beta=0.28, 95% confidence interval (0.018-0.231), P=0.02).

We also found a non-significant negative correlation between Malat1 expression with IL-1β and NIHSS score.

## Discussion

In this study, serum VitD levels in the peripheral blood of patients with IS showed a negative Pearson correlation with IL-1β and a positive correlation with the expression level of Malat1 lncRNA. Linear regression confirmed the significant positive relationship between Malat1 and VitD levels.

Patients with an NIHSS score>7 showed a significantly higher level of IL-1β and lower expression of Malat1 relative to other patients who had an NIHSS score<6 while we couldn’t detect a significant negative correlation between stroke severity and VitD level. We also found a significant negative correlation between VitD and IL-1β.

Previous studies have also demonstrated the contribution of IL-1β or VitD deficiency in the progression of ischemic injury [17][48][26] and the anti-inflammatory effect of Malat1 following IS [11][12]. These findings are consistent with our results. The downregulation of Malat1 with a significant negative association with stroke severity has been reported in patients with IS [12][14]. We found significant negative Pearson correlations (r=-0.25, P=0.04) between Malat1 expression and NIHSS score. Ren et al. reported a negative correlation between Malat1 expression and pro-inflammatory factors expression (CRP, TNF-α, IL-22, IL-6, and IL-8) in patients with IS [12].

We could also detect the significant negative correlation between Malat1 and IL-1β levels. This result may reinforce the anti-inflammatory role of lncRNA Malat1 after stroke which has been reported by previous studies [11][49].

The protective roles of lncRNA Malat1 in cerebrovascular diseases have been reported through activating phosphatidylinositol 3-kinase (PI3K) [50] via inhibition of pro-apoptotic or pro-inflammatory factors [51][52]. Nowrouzi et al.reported a significant decrease in the level of Malat1 and CD36 in peripheral blood mononuclear cells of participants with vitamin D deficiency which was accompanied by a significantly higher plasma level of IL-6, IL-10, and IL-22 [53].

Additionally, the significant positive relationship between Malat1 and VitD levels in our patients may lead to the identification of a novel mechanism for its anti-inflammatory effect in the future.

Evan et al. reported that the expression of IL-1β, IL-6, TGF-β, IL-23a, and NADPH oxidase-2 was decreased after ischemic stroke in the brains of mice supplemented with 1,25-VitD3 and also demonstrated that expression of the 1-α-hydroxylase as a vitamin D-activating enzyme was decreased, while expression of 24-hydroxylase (vitamin D inactivating enzyme), was increased in brain and spleen after a stroke [37].

Thus, the active form of vitamin D in the brain may decrease after stroke.

The negative correlation between Malat1 downregulation and VitD level after stroke leads us to the hypothesis that the reduction of the active form of VitD in the ischemic brain may be due to a decrease in a lncRNA such as Malat1.

Several mechanisms have been investigated for the anti-inflammatory effects of vitamin D, such as regulation of the immune system [54], cytokine release [55], inhibiting nuclear factor kappa-B (NFκB) activity [56], and up-regulating MKP5 [57]. It seems that the Malat1 upregulation appears to be a promising approach to increase the anti-inflammatory mediated effect of VitD.

The novelty of this study relates to the correlation between these parameters (Malat1, IL-1β, and VitD) in the peripheral blood of patients with IS during the first 24 hours after stroke. Understanding the strong correlation between VitD and various cytokines or lncRNAs produced during a stroke may be important for the medical management of stroke severity in patients and will provide the information about attenuation of ischemic damage, especially its anti-inflammatory role.

These correlations need to be confirmed by further studies with a larger sample size along with full transcriptome analysis. Also, by further research, the precise molecular mechanisms of Malat1 and its correlations with VitD and IL-1β in the pathogenesis of IS must be investigated.

## Conclusion

We evaluated the associations of Malat1 expression with VitD level, and IL-1β on the peripheral blood of IS patients 0-24 h after stroke onset for the first time. A significant positive Pearson correlation was detected between VitD and Malat1 expression which was confirmed by stepwise regression analysis. However, we need to study these correlations with a larger sample size, to reach a strong and reliable association between Malat1 and VitD levels in IS patients.

## Acknowledgments

This work was supported by Shiraz University of Medical Sciences (IR.SUMS.REC.1398. with grant agreement No. 17988).

## Conflict of interest

The authors declare no competing interests.
